# The effect of the alpha-specific PI3K inhibitor alpelisib combined with anti-HER2 therapy in HER2+/PIK3CA mutant breast cancer

**DOI:** 10.3389/fonc.2023.1108242

**Published:** 2023-07-04

**Authors:** Maria Letizia Cataldo, Pietro De Placido, Daniela Esposito, Luigi Formisano, Grazia Arpino, Mario Giuliano, Roberto Bianco, Carmine De Angelis, Bianca Maria Veneziani

**Affiliations:** ^1^Department of Clinical Medicine and Surgery, University of Naples Federico II, Naples, Italy; ^2^Department of Molecular Medicine and Medical Biotechnology, University of Naples Federico II, Naples, Italy

**Keywords:** alpelisib, *PIK3CA*, HER2, breast cancer, resistance, *AK1RC1*

## Abstract

**Background:**

HER2 is amplified or overexpressed in around 20% of breast cancers (BC). HER2-targeted therapies have significantly improved the prognosis of patients with HER2+ BC, however, *de novo* and acquired resistance to anti-HER2 treatment is common. Activating mutations in the *PIK3CA* gene are reported in ∼30% of HER2+ BC and are associated with resistance to anti-HER2 therapies and a poor prognosis. Here, we investigated the *in vitro* and *in vivo* antitumor efficacy of the alpha-specific PI3K inhibitor alpelisib alone or in combination with anti-HER2 therapy using a panel of HER2+ BC cell lines. We also generated models of acquired resistance to alpelisib to investigate the mechanisms underlying resistance to alpha-specific PI3K inhibition.

**Materials and methods:**

*PIK3CA* mutant (HCC1954, KPL4 and JMT1) and *wild-type* (BT474 and SKBR3) HER2+ BC cell lines were used. The HCC1954 and KPL4 cells were chronically exposed to increasing concentrations of alpelisib or to alpelisib + trastuzumab in order to generate derivatives with acquired resistance to alpelisib (AR) and to alpelisib + trastuzumab (ATR). The transcriptomic profiles of HCC1954, KPL4 and their AR and ATR derivatives were determined by RNA sequencing. Cell growth was assessed by MTT assay. Changes in the protein levels of key PI3K pathway components were assessed by Western blotting. Gene expression, cellular and patients’ data from the Cancer Dependency Map (DepMap) and KMPlot datasets were interrogated.

**Results:**

HER2+ BC cell lines harboring activating mutations in *PIK3CA* were less sensitive to single or dual anti-HER2 blockade compared to *PIK3CA* wild-type cells. Alpelisib treatment resulted in dose-dependent inhibition of the growth of cells with or without *PIK3CA* mutations and enhanced the antitumor efficacy of anti-HER2 therapies *in vitro*. In addition, alpelisib greatly delayed tumor growth of HCC1954 xenografts *in vivo*. Functional annotation of the significantly differentially expressed genes suggested the common activation of biological processes associated with oxidation reduction, cell proliferation, immune response and RNA synthesis in alpelisib-resistant models compared with native cells. Eight commonly upregulated genes (log2 fold-change >1, False Discovery Rate [FDR] <0.05) in models with acquired resistance to alpelisib or alpelisib + trastuzumab were identified. Among these, *AKR1C1* was associated with alpelisib-resistance *in vitro* and with a poor prognosis in patients with HER2+ BC.

**Conclusions:**

Our findings support the use of an alpha-selective PI3K inhibitor to overcome the therapeutic limitations associated with single or dual HER2 blockade in *PIK3CA*-mutant HER2+ breast cancer. Future studies are warranted to confirm the potential role of candidate genes/pathways in resistance to alpelisib.

## Introduction

The human epidermal growth factor receptor-2 (HER2) is amplified or overexpressed in around 15-20% of breast cancers. HER2-positive breast cancer is an aggressive disease historically associated with high relapse and mortality rates. Fortunately, the development of HER2-targeted monoclonal antibodies such as trastuzumab and pertuzumab has significantly improved the outcomes of patients with HER2-positive breast cancer. Besides trastuzumab and pertuzumab, other agents have recently expanded the treatment landscape of HER2-positive advanced disease, namely the oral tyrosine kinase inhibitors tucatinib and neratinib and the antibody drug conjugates trastuzumab emtansine (T-DM1) and trastuzumab deruxtecan (T-DXd). The advent of these agents was associated with a further improvement in survival outcomes (1).

However, *de novo* and acquired resistance to anti-HER2 treatments is common and remains a major clinical problem. Several mechanisms underlying resistance to anti-HER2 therapy have been suggested (2–5). One mechanism is the constitutive activation of the phosphoinositide 3-kinase (PI3K) pathway. The latter is a key downstream signaling pathway of HER2 that mainly occurs through mutations or amplification of the *PIK3CA* gene encoding the p110α catalytic subunit of PI3K, loss of the phosphatase and tensin homologue (PTEN), and mutations of the AKT gene encoding a serine/threonine-specific protein kinase. Activating mutations in *PIK3CA* are reported in around 30% of HER2+ breast cancers and are associated with aggressive tumor behavior and poor treatment outcomes. In the phase III CLEOPATRA trial the progression-free survival after first-line dual HER2 blockade with trastuzumab plus pertuzumab was shorter in patients with *PIK3CA* mutant breast cancer than in patients with wild-type (WT) *PIK3CA* tumors (6).

Activating *PIK3CA* mutations have also been associated with a lower response rate to neoadjuvant anti-HER2 therapy in patients with early-stage HER2+ breast cancer (7, 8). In the preclinical setting, HER2+ breast cancer cell lines harboring *PIK3CA* mutations are resistant to trastuzumab (9, 10) and retain AKT phosphorylation despite treatment (11). *PIK3CA* mutations also confer resistance to trastuzumab, pertuzumab, and/or the HER2 tyrosine kinase inhibitor (TKI) lapatinib *in vivo (* 12). Of note, in HER2+/*PIK3CA* mutant preclinical models, PI3K inhibitors enhance the efficacy of anti-HER2 therapies and delay or overcome resistance to these drugs. Given the key role played by the PI3K pathway in cancer, several PI3K inhibitors have been developed, but few have received regulatory approval due to limited activity and/or a toxic effect profile (13–16). Recently, the alpha-specific PI3K inhibitor alpelisib (BYL719), in addition to fulvestrant, led to clinically meaningful results. Tolerability was acceptable in patients with estrogen receptor (ER)+/HER2-, *PIK3CA* mutated, advanced or metastatic breast cancer. In this study, we evaluated the efficacy of alpelisib in HER2+/*PIK3CA* mutant breast cancer using *in vitro* and *in vivo* models. We also developed HER2+/*PIK3CA* mutant breast cancer models of acquired resistance to alpelisib to investigate the molecular mechanisms underlying resistance.

## Material and methods

### Cell lines, establishment of resistant lines, and reagents

HER2+ breast cancer cell lines SKBR3 (ATCC^®^ HTB-30™), BT474 (ATCC^®^ HTB-20™), and HCC-1954 (ATCC^®^ CRL-2338™) were obtained from the American Type Culture Collection (ATCC). KPL4 and JMT1 were obtained from the Laboratory of Roberto Bianco, Department of Clinical Medicine and Surgery, University of Naples “Federico II”, Italy. SKBR3, HCC-1954, KPL4 and JIMT1 cells were grown in RPMI-1640 medium (Gibco, Invitrogen, Milan, Italy) supplemented with 10% fetal bovine serum (FBS; Gibco, Invitrogen, Milan, Italy), 100 U/mL penicillin, and 100 μg/mL streptomycin. BT474 cells were grown in DMEM medium (Gibco, Invitrogen, Milan, Italy) supplemented with 10% fetal bovine serum (FBS; Gibco, Invitrogen, Milan, Italy), 100 U/mL penicillin, and 100 μg/mL streptomycin. All cells were maintained under 5% CO2 in a humidified incubator at 37°C. Cells were routinely tested for mycoplasma using MycoFluor (Mycoplasma Detection Kit MycoAlert, M7006, Invitrogen, Milan, Italy). Trastuzumab and pertuzumab were purchased from SelleckChemed (Munich, Germany). Alpelisib was provided by Novartis Pharma (Basel, Switzerland). All experiments were conducted within two months of thawing of the acquired cells. Alpelisib-Resistant (AR) and Alpelisib-Trastuzumab-Resistant (ATR) derivatives were generated by exposing HCC1954 and KPL4 cell lines to gradually increasing doses of alpelisib (from 0.125μM to 1μM) or concomitant alpelisib (from 0.125μM to 1μM) plus trastuzumab (from 1μg/ml to 10μg/ml).

### Cell growth assays

Four to eight thousand cells per well were seeded in 24-well plates and treated with the reported concentrations of trastuzumab and pertuzumab for 7 days and analyzed with the 3-(4,5-dimethylthiazol-2-yl)-2,5-diphenyltetrazoliumbromide (MTT) assay according to the manufacturer’s instructions (Sigma–Aldrich, Italy) as previously described (17, 18). Experiments were performed three times; values represent means ± SEM from quadruplicate samples for each treatment. The percentage of absorbance of treated samples versus untreated is reported as a percentage of viable cells/controls. IC50 values were generated through GraphPad Prism v8.01 using the log (inhibitor) versus response-variable slope (four parameters) model as previously described (19).

### RNA interference

HER2+ breast cancer cells were transfected with Lipofectamine RNAiMAX^®^ (Invitrogen) and 20 nM siRNA [siControl - OriGene cat.#; siAKR1C1#1-2-3 OriGene_SR301163], and seeded in 24-well multiwells (for cell growth assays) or 60 mm plates (for immunoblot assays) and incubated at 37°C, 5% CO_2_ for 24 hours. The next day, the transfection medium (without antibiotics/antifungals) was replaced with the complete culture medium in the presence or absence of the drug. The culture medium ± drugs was replaced every 72 hours. On the sixth day post-transfection, the cells were analyzed using the MTT method. For immunoblot assays, cells were harvested and lysed 72 hours post-transfection.

### Immunoblotting assay

The cells were plated, allowed to grow for 24 h before treatments and then treated with the drugs for 48h. All cells were maintained under 5% CO_2_ in a humidified incubator at 37°C. The cell pellet was obtained after treatment with trypsin and centrifugation for 5 minutes at 1300 rpm. Protein preparations from cells were obtained by lysing samples with RIPA lysis buffer (ChemCruz, Dallas, TX). The samples were incubated on ice for 30 minutes and then centrifuged at 13200 rpm for 20 minutes at 4°C. Protein concentration was measured by the Bio-Rad Protein Assay (Bio-Rad, Milan, Italy). Twenty-five–microgram aliquots were electrophoresed through 8–10% SDS polyacrylamide gels. Prestained molecular weight standards were from Bio-Rad. The membrane was stained with Ponceau S (Sigma–Aldrich, Italy) to evaluate the success of transfer. Free protein binding sites were blocked with nonfat dry milk and Tween-20/TBS solution (5%). The membranes were washed, stained with specific primary antibodies (Ab) and then with secondary antisera, conjugated with horseradish peroxidase (1:3.000; Santa Cruz Biotechnology, Inc., Santa Cruz, CA). The following primary antibodies were used: Ab anti-phosphorylated (p-) HER2 (CST#2243, 1:1000), Ab anti-PTEN (CST #9559, 1:1000), Ab anti-AKT (CST#9272, 1:1000), Ab anti-p-AKT S473 (CST#9271, 1:1000), Ab anti-S6K, Ab anti-p-S6K T389 (CST#9205, 1:1000), Ab anti-beta-actin (CST#4970, 1:5000). The luminescent signal was visualized with the ECL Western blotting detection reagent kit (Bio-Rad, Milan, Italy) and detected by light-sensitive films (FUJIFILM Super RX-N 100 NIF).

### RNA sequencing and analysis

KPL4 and HCC1954 cells were plated in complete medium and after 24 h treated with vehicle (DMSO), 1 µM alpelisib or 1 µM alpelisib + 10 µg/ml trastuzumab for 48 hours. KPL4-AR and HCC1954-AR cells were plated in complete culture medium in the presence of 1 µM alpelisib. KPL4-ATR and HCC1954-ATR cells were plated in complete culture medium in the presence of 1 µM alpelisib + 10 µg/ml trastuzumab. Total RNA of the HCC1954 and KPL4 cell lines was extracted with RNeasy Mini Kit (Qiagen, Germany). The RNA-seq libraries were prepared using the TruSeq RNA Sample Preparation Kit (Illumina) and the Agilent Automation NGS system per manufacturers’ instructions. Samples were sequenced on an Illumina HiSeq platform with paired-end 150 bp reads. Sequence reads were trimmed to remove possible adapter sequences and nucleotides with poor quality using Trimmomatic v.0.36. The trimmed reads were mapped to the Homo sapiens GRCh38 reference genome available on ENSEMBL using the STAR aligner v.2.5.2b. The STAR aligner is a splice aligner that detects splice junctions and incorporates them to help align the entire read sequences. BAM files were generated as a result of this step. Unique gene hit counts were calculated by using featureCounts from the Subread package v.1.5.2. The hit counts were summarized and reported using the gene_id feature in the annotation file. Only unique reads that fell within exon regions were counted. If a strand-specific library preparation was performed, the reads were strand-specifically counted. After extraction of gene hit counts, the gene hit counts table was used for downstream differential expression analysis. Using DESeq2, a comparison of gene expression between the groups of samples was performed. The Wald test was used to generate p-values and log2 fold changes. Genes with an adjusted p-value <0.05 and absolute log2 fold change > 1 were called as differentially expressed genes for each comparison. A gene ontology analysis (20) was performed on the statistically significant set of genes by implementing the GeneSCF v.1.1-p2 software. The goa_human GO list was used to cluster the set of genes based on their biological processes and determine their statistical significance. The GSEA software (Broad Institute, Massachusetts, USA) and MSigDB v6.2 Hallmark gene sets collection were used to calculate enrichment scores.

### Survival analysis

Kaplan–Meier survival curves were generated using an online meta-analysis tool for public microarray datasets (21). This integrative data analysis tool was employed to evaluate the prognostic value of the following genes: *ARL4A*, *SNRPG*, *ODC1*, *AKR1C1*, *TRIM16L*, *AKR1C2*, *GLB1L2*, and *BDH1*. Kaplan–Meier plots were generated after averaging the probes. The selected clinical cases were HER2+ (defined by immunohistochemistry) breast tumors divided into two groups (high and low) by the median expression value of each gene. P values were determined by the log-rank test.

### *In vivo* experiments

HCC 1954 cells were maintained as described above. Ovariectomized 5- to 6-week-old athymic mice (Charles River, Italy) received a subcutaneous injection into the right flank of 1×10^6^ HCC1954 cells suspended in Matrigel + RPMI-1640 (1:1). When the volume of the cell xenografts reached ~150–200 mm^3^, mice were randomly assigned (n=10 per group) to receive vehicle, 30 mg/kg trastuzumab (via intraperitoneal administration twice weekly), 30 mg/kg alpelisib (administered orally *via* gavage, daily) or the combination of 30 mg/kg trastuzumab plus 30 mg/kg alpelisib. Alpelisib was dissolved in PEG400/acidified water (0.1 N HCl, pH 3.5; 20/80, v/v). Tumor dimensions and body weights were recorded twice weekly starting on the first day of treatment. The tumor volume was calculated according to the formula π/6 × (larger diameter) × (smaller diameter)^2^. The *in vivo* antitumor activity was measured using the rate-based T/C as previously described (22). All animal experiments were performed according to protocols approved by the Animal Care and Use Committee of Federico II University.

### Statistical analysis

Statistical analyses were performed using GraphPad Prism v9.01. Quantitative data are shown as mean ± SEM. A p-value <0.05, calculated by two-tailed Student’s t-test, ANOVA with Bonferroni’s or Dunnett/Tukey’s post-hoc tests, or Pearson/Spearman correlation coefficient test was considered as statistically significant.

## Results

### Efficacy of alpelisib alone or in combination with anti-HER2 monoclonal antibodies in HER2+ breast cancer cell lines

**Figure 1:**
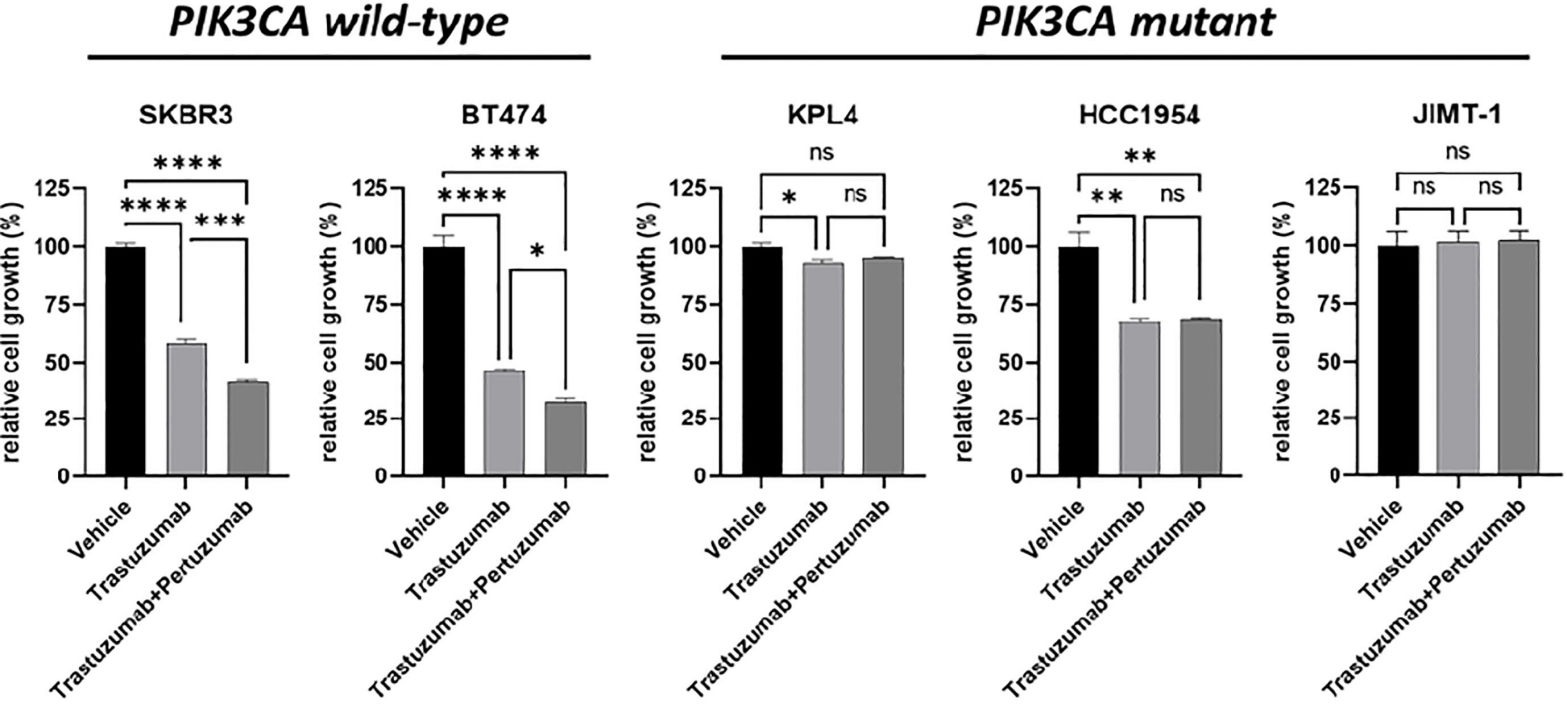
Cell growth inhibition *in vitro* of HER2+ breast cancer cell lines treated with monoclonal anti-HER2 antibodies. Cells were plated in 24-well plates and treated with vehicle (DMSO), 10 μg/ml trastuzumab, or 10 μg/ml trastuzumab + 10 μg/ml pertuzumab. Cell growth was assessed after 6 days. Data were normalized to vehicle. Data represent means ± SEM. *p-value <0.05, **p-value <0.01; ***p-value <0.001; ****p-value <0.0001; ns, not significant according to multiple t test with Bonferroni correction.

**Figure 2:**
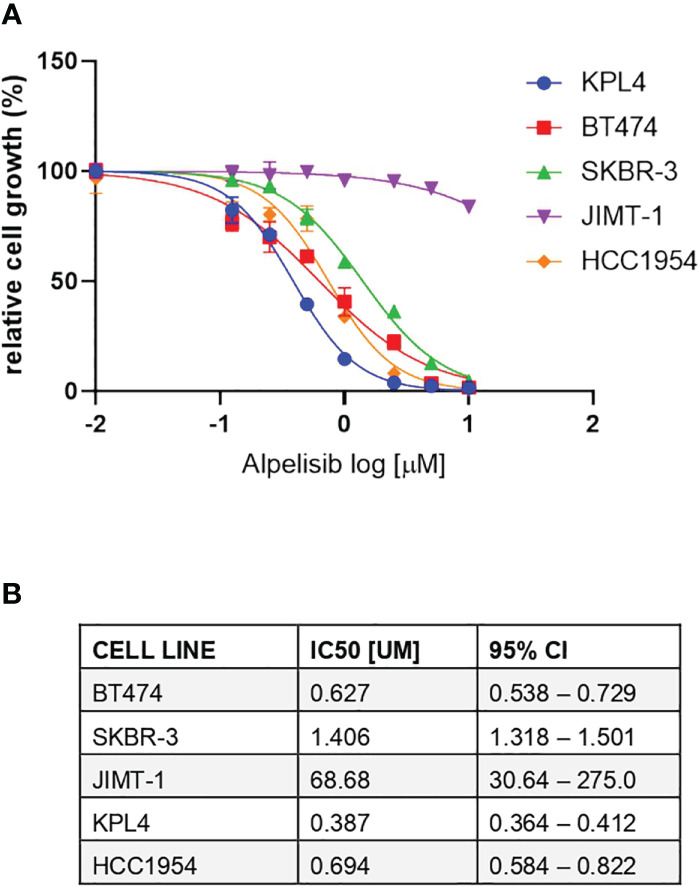
Responses of HER2+ breast cancer cell lines to the alpha-specific PI3K inhibitor alpelisib. **(A)**, Cells were plated in 24-well plates and treated with increasing doses of alpelisib. Cell growth was assessed after 6 days. Data were analyzed by GraphPad Prism (version 9.0) to generate drug response curves and relative IC50 values using the log (inhibitor) versus response-variable slope model (bars, SEM) with normalization of data defining the biggest number in each dataset as 100% and the smallest number in the same dataset as 0%. **(B)**, Estimated IC50 values of alpelisib in HER2 + breast cancer cell lines. CI, confidence of interval.

**Figure 3:**
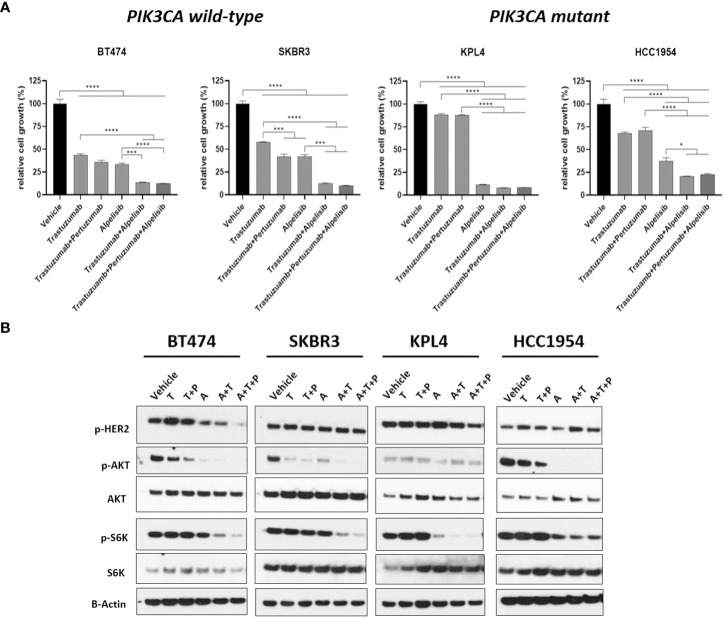
The alpha-specific PI3K inhibitor alpelisib increases the efficacy of anti-HER2 therapy in HER2+ breast cancer cell lines. **(A)**, Cells were plated in 24-multiwell and treated with vehicle (DMSO), 10μg/ml trastuzumab, 10μg/ml trastuzumab + 10μg/ml pertuzumab, 1μM alpelisib, 1μM alpelisib + 10μg/ml trastuzumab, 1μM alpelisib + 10μg/ml trastuzumab + 10μg/ml pertuzumab. Cell growth was assessed after 6 days. Data were normalized to vehicle. Data represent means ± SEM. *p-value <0.05; ***p-value <0.001; ****p-value <0.0001 according to multiple t test with Bonferroni correction. **(B)**, Western blot analysis of total and phosphorylated proteins of the HER2/PI3K pathway signaling. Cells were plated in 6-multiwell and treated with vehicle (DMSO), 10μg/ml trastuzumab (T), 10μg/ml trastuzumab + 10μg/ml pertuzumab (T+P), 1μM alpelisib (A), 1μM alpelisib + 10μg/ml trastuzumab (A+T), 1μM alpelisib + 10μg/ml trastuzumab + 10μg/ml pertuzumab (A+T+P).

**Figure 4:**
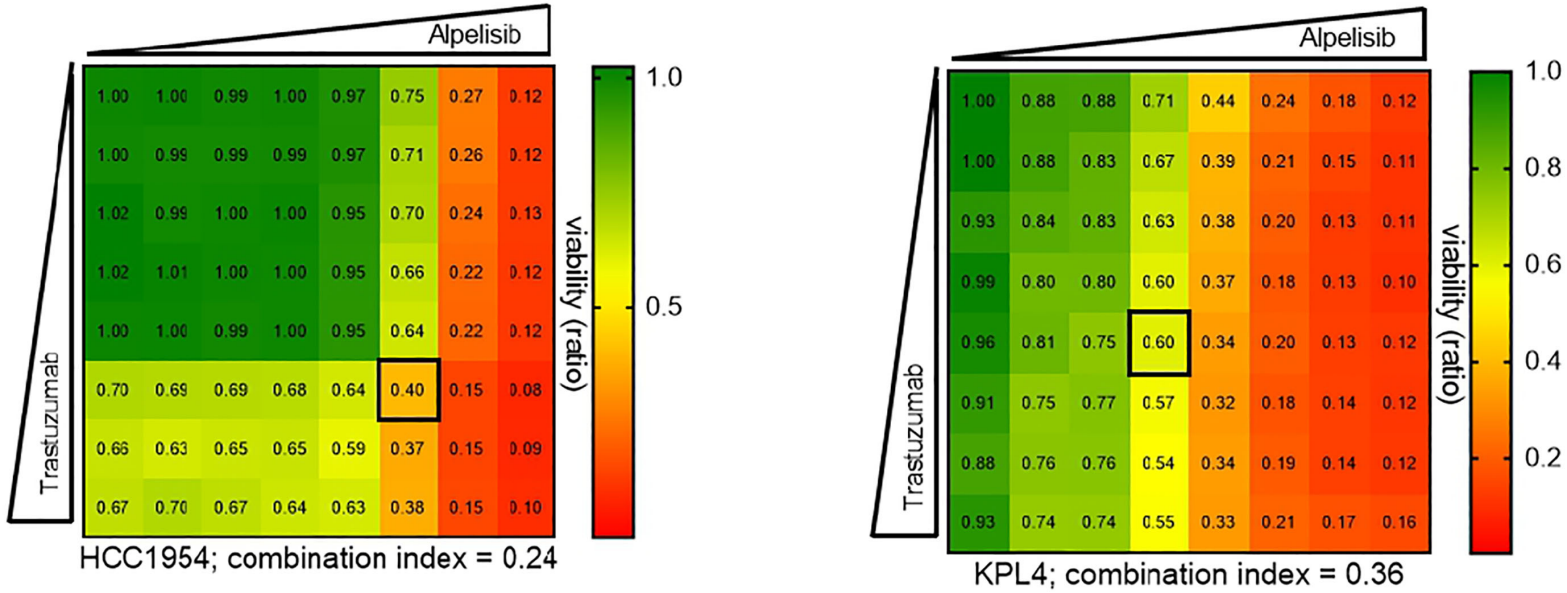
Pharmacological synergy between alpelisib and trastuzumab in HER2+/*PIK3CA* mutant breast cancer cell lines. HCC1954 and KPL4 cells were seeded in 96 well plate treated with increasing concentrations of each drug alone or in combination (up to 10μM alpelisib and 100μg/ml trastuzumab) for 7 days. Combination indices were determined using the Chou-Talalay test by CompuSyn software. Numbers inside each box indicate the ratio of viable treated cells to untreated cells, from three independent experiments. Synergy is defined as combination index (CI)<1, an additive effect as CI=1, and antagonism effect as CI>1.

We selected a panel of *PIK3CA* mutant (KPL4, JMT1, and HCC1954) and *PIK3CA* WT (SKBR3 and BT474) HER2+ breast cancer cell lines. The estrogen receptor (ER), the progesterone receptor (PR), and the *PIK3CA* status of each cell line are shown in **Supplementary Table 1**. First, we investigated the efficacy of the anti-HER2 monoclonal antibodies trastuzumab and pertuzumab in our panel of HER2+ breast cancer cell lines (**Figure 1**). In line with the results of previous studies (3, 11), trastuzumab or the combination of trastuzumab plus pertuzumab inhibited the growth of BT474, SKBR3 and HCC1954 cell lines. On the contrary, the anti-HER2 monoclonal antibodies had no effect or only a modest inhibitory effect on JIMT1 and KPL4 cell lines, respectively. We next assessed the efficacy of alpelisib in our models and found that Alpelisib treatment resulted in a dose-dependent inhibition of cell growth of KPL4, HCC1954, SKBR3 and BT474 cell lines but not of JIMT1 cells that had a complete *de novo* resistance to the drug (**Figure 2A**). The calculated alpelisib IC50 values are reported in **Figure 2B**. The KPL4 cell line was the most alpelisib-sensitive, while the intrinsic alpelisib-resistant JIMT1 model was excluded from subsequent experiments. We next investigated the efficacy of anti-HER2 monoclonal antibodies (trastuzumab or trastuzumab plus pertuzumab), alpelisib, or their combinations in our panel of HER2+ breast cancer cell lines. Specifically, KPL4, HCC1954, SKBR3 and BT474 cell lines were subjected to vehicle (DMSO, Sigma–Aldrich, Italy), 10μg/ml trastuzumab, 10μg/ml trastuzumab + 10μg/ml pertuzumab, 1μM alpelisib, 1μM alpelisib + 10μg/ml trastuzumab, and the triplet combination of 1μM alpelisib + 10μg/ml trastuzumab + 10μg/ml pertuzumab (**Figure 3**). We observed that alpelisib monotherapy was more effective than trastuzumab or trastuzumab + pertuzumab in inhibiting cell growth in the *PIK3CA* mutant KLP4 and HCC1954 cell lines (growth inhibition >90% and >70%, respectively). Among *PIK3CA* WT models, no differences in cell growth inhibition were observed between trastuzumab or trastuzumab + pertuzumab vs. alpelisib treatments in BT474 cells, differently, alpelisib had a higher inhibitory effect than trastuzumab whereas trastuzumab + pertuzumab in the SKBR3 cells did not. Interestingly, the addition of alpelisib to the anti-HER2 therapy resulted in a more pronounced growth inhibition in 3 out of the 4 cell line models (BT474, SKBR3 and HCC1954) (**Figure 3A**). Western blot analysis of selected components of the PI3K pathway revealed a greater reduction of phosphorylated AKT (p-AKT) and ribosomal S6 kinase protein (p-S6K) levels upon treatment with alpelisib plus anti-HER2 therapy (trastuzumab or trastuzumab + pertuzumab) versus alpelisib or anti-HER2 therapy alone in SKBR3 and BT474 cell lines (**Figure 3B**). In *PIK3CA* mutant KPL4 and HCC1954 models, the addition of alpelisib to anti-HER2 therapy resulted in a higher inhibition of p-S6K but not p-AKT protein levels (**Figure 3B**). We also assessed the potential drug interaction between alpelisib and trastuzumab in *PIK3CA* mutant HER2+ breast cell lines using Chou-Talalay method (23), where a combination index (CI) < 1 indicates synergy between drugs. Viability assays using increasing concentrations of alpelisib and trastuzumab demonstrated a pharmacological synergism between these agents in both HCC1954 and KPL4 models (CI=0.24 and CI=0.36, respectively; **Figure 4**).

### HER2 and PI3K blockade in HER2+/*PIK3CA* mutant breast cancer xenografts

**Figure 5:**
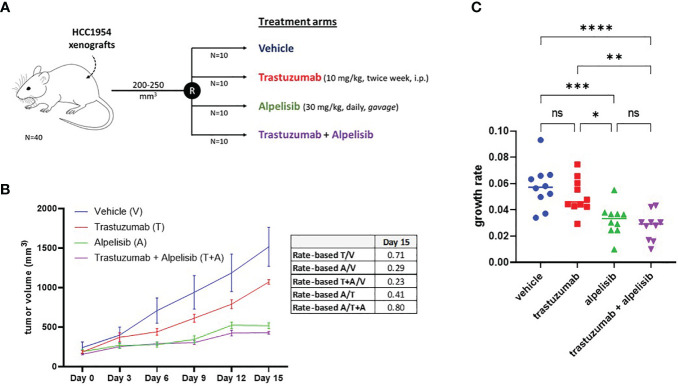
*In vivo* efficacy alpelisib alone or in combination with trastuzumab. **(A)**, Mice were injected with 5 x106 HCC1954 cells and randomized to receive vehicle, trastuzumab, alpelisib, alpelisib + trastuzumab (see Materials and Methods for details). **(B)**, Tumor growth curves obtained by measuring the volumes of the tumors in the study groups. Data points represent mean ± SEM tumor volume. Table reported the rate-based T/C computed using the data up to day 15. **(C)**, Growth rates of HCC1954 xenografts by treatment arm. Each dot represents a different mouse. *P <0.05, **P <0.01, ***P <0.001, ****P <0.0001; ns, not significant according to multiple t test with Bonferroni correction.

The inhibitory activity of alpelisib on tumor growth was evaluated *in vivo* using xenografts of HCC1954 cells. When tumors reached a volume of approximately 200 mm^3^, mice were randomized into the following treatment groups: vehicle (n=10), trastuzumab (n=10), alpelisib (n=10) and trastuzumab + alpelisib (n=10) (**Figure 5**). In the absence of treatment (vehicle), HCC1954 xenografts had a high growth rate, reaching a tumor volume of 1500 mm^3^ in about 15 days. In line with the results of our *in vitro* experiments, tumor growth was moderately slower in tumors treated with trastuzumab than in those treated with vehicle. In addition, tumor growth was slower in alpelisib-treated tumors than in those treated with vehicle. Alpelisib treatment substantially inhibited tumor growth unlike treatment with vehicle or trastuzumab. Finally, the combination of alpelisib plus trastuzumab reduced growth versus the individual drugs, although the difference with the alpelisib treatment arm was not statistically significant (**Figure 5**). Notably, total regression of the tumor was not found in any of the treatment arms. Overall, our data support the potential role of alpelisib for the treatment of HER2+/PI3KCA mutated breast cancers.

### Mechanisms of acquired resistance to alpelisib in HER2+/*PIK3CA* mutant breast cancer

**Figure 6:**
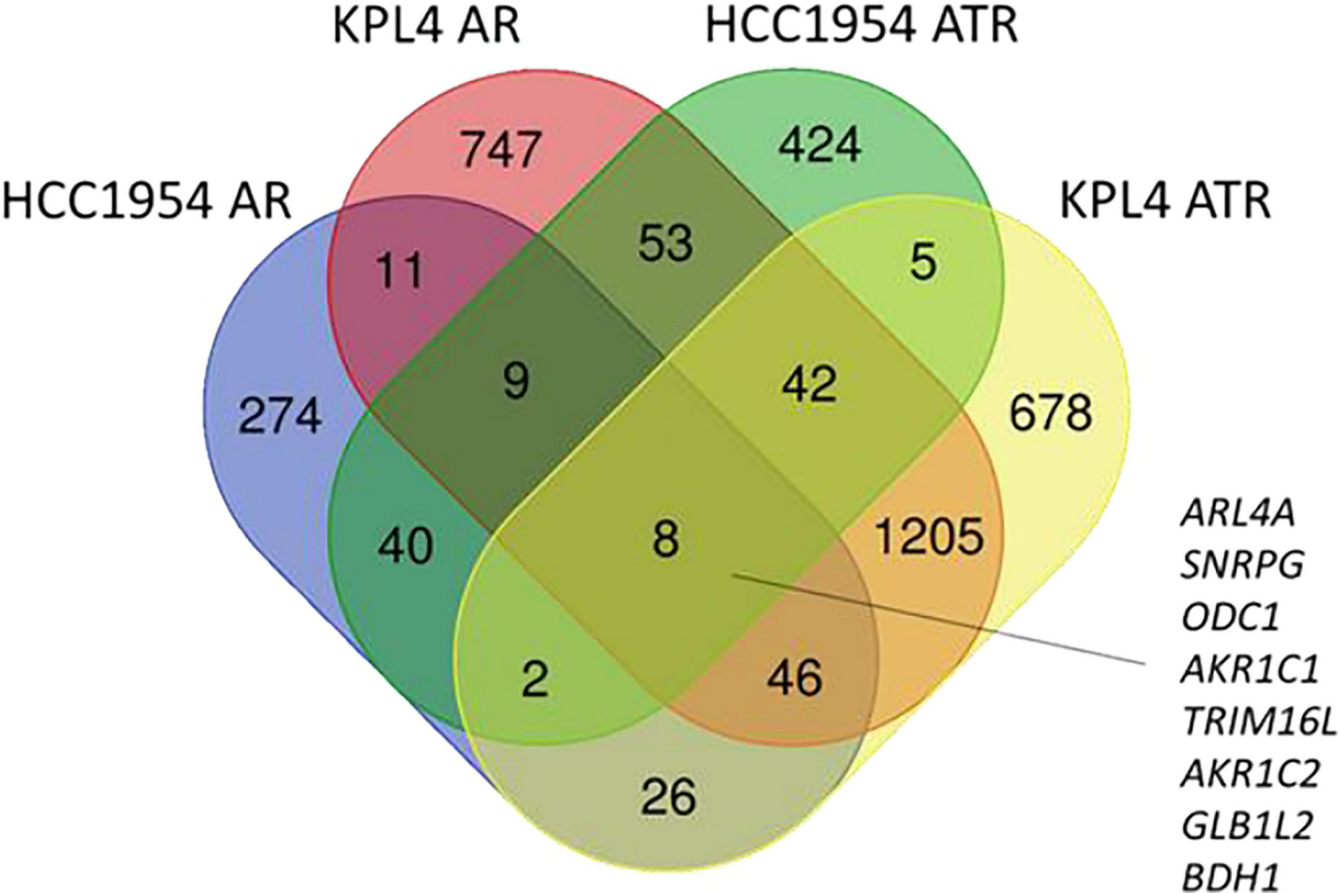
Venn diagram of significantly up-regulated genes in HER2+/*PIK3CA* mutant alpelisib-resistant (AR) and alpelisib + trastuzumab-resistant (ATR) derivatives.

To investigate the molecular mechanisms promoting resistance to alpha-specific PI3K inhibition, we generated derivatives with acquired resistance to alpelisib and to alpelisib plus trastuzumab (ATR) from KPL4 and HCC1954 cells by exposing cells to increasing doses of alpelisib or alpelisib + trastuzumab. Next, we analyzed the transcriptomic profiles of the HCC1954 and KPL4 cell lines treated with short term (48 hours) alpelisib or alpelisib + trastuzumab together with their resistant derivatives in order to identify transcriptional alterations underlying alpelisib resistance. The differentially expressed genes (DEG) in alpelisib-resistant vs sensitive HER2+/*PIK3CA* mutant breast cancer cell lines are reported in **Supplementary Table S1**. Gene ontology analysis of the DEG between sensitive vs resistant cells showed a common enrichment for biological processes associated with oxidation reduction (“oxidation reduction process”), proliferation and cell division (“cell division”, “cell proliferation”), immune response (“inflammatory response”, “innate immune response”) and RNA synthesis (“positive regulation of transcription, DNA template”) in AR and ATR models (**Supplementary Figure S1**). We next focused on the significantly up-regulated genes in AR and ATR models. Globally 8 genes (*ARL4A*, *SNRPG*, *ODC1*, *AKR1C1*, *TRIM16L*, *AKR1C2*, *GLB1L2*, and *BDH1*) were found to be commonly overexpressed (log2 fold-change >1, adjusted p-value <0.05) in our resistant models (**Figure 6**).

**Figure 7:**
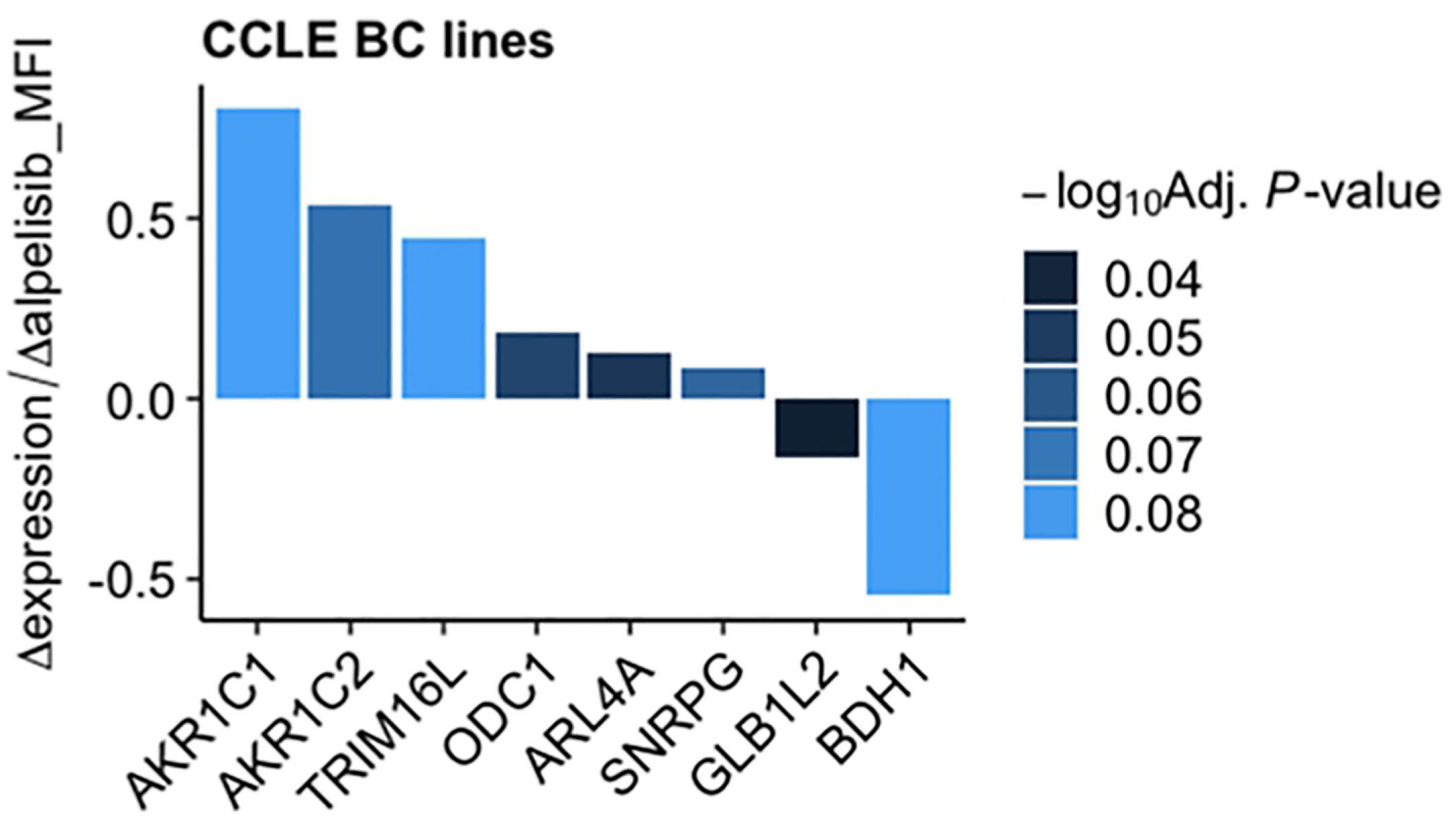
Association between gene expression and *in vitro* sensitivity to alpelisib in breast cancer cell lines. Bars indicate fold-change between mRNA expression of the 8 genes commonly overexpressed in our alpelisib resistant models and *in vitro* cell viability upon treatment with alpelisib measured as median fluorescence intensity (MFI) in 26 breast cancer cell lines. Data were downloaded from the “*Cancer Dependency Map Portal*” (https://depmap.org/portal/achilles/).

**Figure 8:**
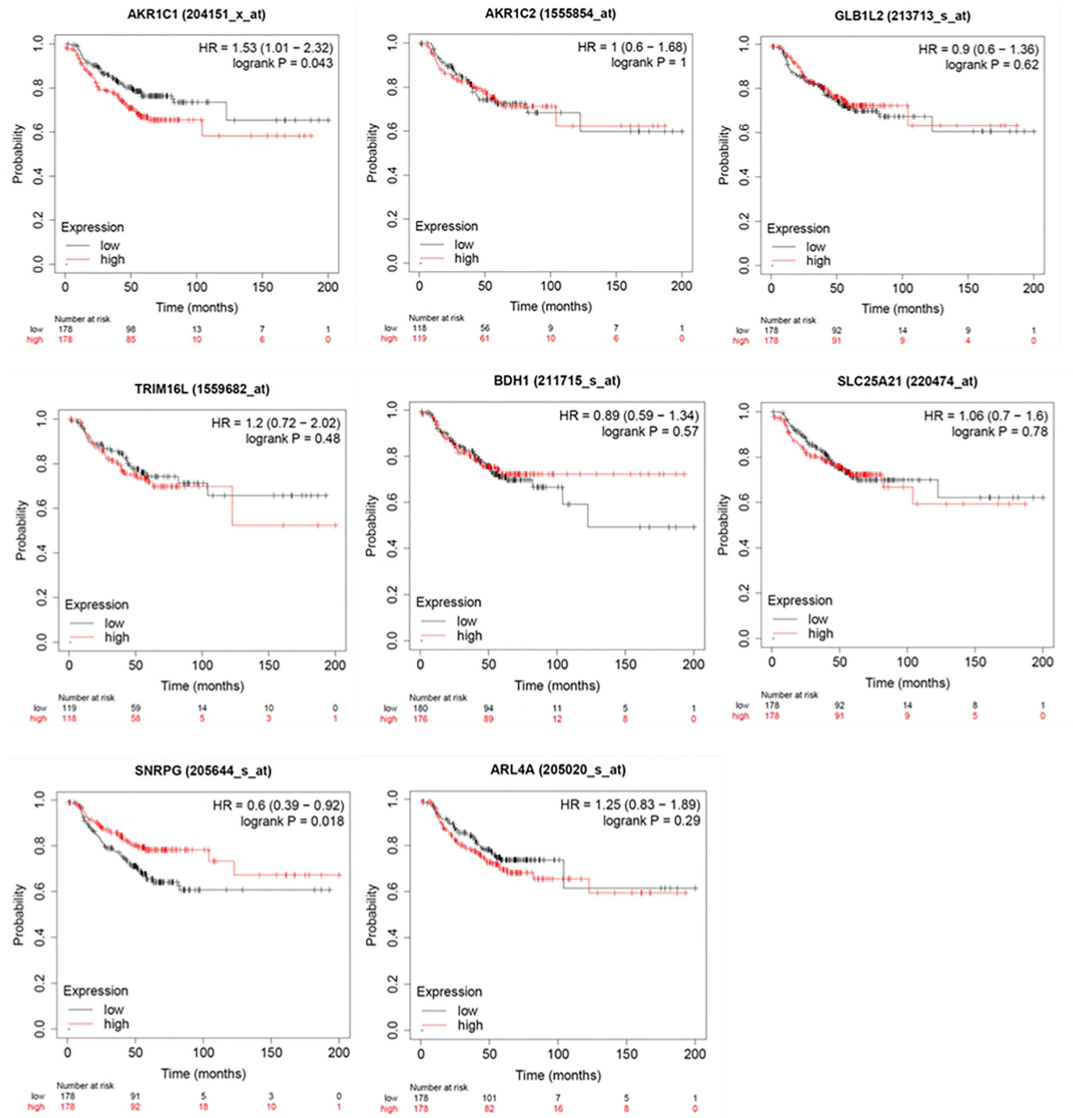
Kaplan-Meier curves showing relapse-free survival (RFS) of HER2+ breast cancer patients. Log-rank test was used for comparing two groups with high (red) and low (black) expression of *ARL4A*, *SNRPG*, *ODC1*, *AKR1C1*, *TRIM16L*, *AKR1C2*, *GLB1L2*, and *BDH1* genes. Number of patients at risk are indicated below each panel. HR, hazard ratio.

**Figure 9:**
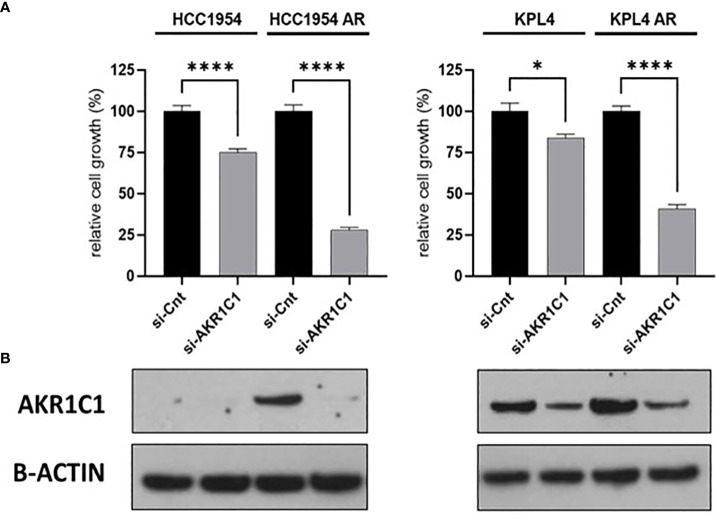
Effect of *AKR1C1* siRNA knockdown in parental and alpelisib-resistant HER2+/*PIK3CA* mutant breast cancer cell lines. **(A)**, HCC1954 and KPL4 parental and alpelisib-resistant (AR) cell lines were transfected with siRNA targeting the AKR1C1 or siRNA control (si-Cnt). Culture medium was replaced the next day with regular medium or alpelisib-containing medium, and replaced again at 4 days. Cell growth was assessed at day 6. Data were normalized to si-Cnt transfection. Data represent means ± SEM. *p-value <0.05; ****p-value <0.0001 according to multiple t test with Bonferroni correction. **(B)**, Protein levels of AKR1C1 in HER2+/PIK3CA mutant breast cancer cell lines transfected with siRNA targeting siRNA control or against AKR1C1 were tested by Western blot.

To investigate the impact of these genes in determining alpelisib sensitivity, we used genomic and cellular data from the Broad Institute’s “*Cancer Dependency Map Portal*” (https://depmap.org/portal/achilles/). In particular, we analyzed the association between the expression levels of each gene and the *in vitro* growth inhibitory effect of alpelisib in 26 breast cancer cell lines. This analysis revealed that the expression of 6 genes (*AKR1C1*, *AKR1C2*, *TRIM16L*, *ODC1*, *ARL4A* and *SNRPG*) negatively correlates with alpelisib sensitivity (**Figure 7**). We also investigated the prognostic value of these genes by evaluating their expression in primary HER2+ breast tumors. To conduct this analysis, we used the publicly available clinical and gene expression data from the “Kaplan-Meier Plotter” database (https://kmplot.com/analysis/). Among the 8 genes analyzed, only *AKR1C1* and *SNRPG* had a significant prognostic value. In detail, patients with HER2+ breast cancer and high expression of *AKR1C1* had a higher risk of recurrence than did patients with low *AKR1C1* expression (Hazard Ratio [HR]=1.53, 95% Confidence Interval [CI] 1.01-2.32, p-value = 0.043). Conversely, in the same patient population, elevated expression of *SNRPG* was associated with a better prognosis (HR=0.6, 95% CI 0.39-0.92, p-value = 0.018) (**Figure 8**). Lastly, we examined the effects of changes in *AKR1C1* expression on cell growth of alpelisib sensitive and resistant lines (HCC1954, HCC1954 AR, KPL4 and KPL4 AR). *AKR1C1* knock-down by siRNA resulted in a significant reduction of cell growth of parental cells (range 20-25%). However, in HCC1954 and KPL4 AR models, which had higher *AKR1C1* protein levels versus parental cells, *AKR1C1* knock-down resulted in a more pronounced cell growth inhibition (range 70-75%). We also found that knockdown of *AKR1C1* enhanced the effect of alpelisib in inhibiting cell growth in both HCC1954 and KPL4 cell lines (**Supplementary Figure S2**). Overall, these data indicate that *AKR1C1* may play *a* potential role in determining the sensitivity to alpelisib and sustaining cell growth of models with acquired resistance to alpelisib (**Figure 9**).

## Discussion

Herein, we examined the impact of *PIK3CA* activating mutations on the efficacy of anti-HER2 therapies and explored the therapeutic potential of alpha-selective PI3K pharmacological inhibition in HER2+/*PIK3CA* mutant breast cancer using pre-clinical *in vitro* and *in vivo* models. Accumulating evidence supports the prognostic and predictive value of PIK3CA mutations in patients with HER2+ breast cancer treated with anti-HER2 therapies in both the adjuvant and the metastatic settings. In the phase III CLEOPATRA study, patients with HER2 + advanced breast cancer and the *PIK3CA* activating mutations had a worse prognosis versus patients with HER2 +/PIK3CA wild-type tumors, regardless of the anti-HER2 treatment administered (trastuzumab or trastuzumab + pertuzumab) (6).

In the early-stage setting, a recent study combined data from individual patients (patient-level data) enrolled in five clinical trials of neoadjuvant therapy with trastuzumab, lapatinib, or the combination of trastuzumab and lapatinib and analyzed the association between the *PIK3CA* mutational status and the pathological complete response (pCR) rates (10). The results demonstrated that patients with HER2+/PIK3CA mutated breast cancer achieved a significantly lower pCR rate than patients with HER2+/PIK3CA wild-type breast cancer (10). However, no significant association was found between PIK3CA mutations and resistance to anti-HER2 antibodies in other clinical trials (10, 24). Our data confirm that the presence of *PIK3CA* mutations is associated with reduced sensitivity to both trastuzumab monotherapy and the dual anti-HER2 blockade with trastuzumab plus pertuzumab in HER2+ breast cancer preclinical models. Therefore, anti-HER2 therapy alone may not be adequate treatment for those patients with HER2+ breast cancer harboring a *PIK3CA*-activating mutation that are associated with a constitutive activation of the PI3K/AKT/mTOR pathway. These patients could potentially benefit from the use of inhibitors of the PI3K/AKT/mTOR signaling pathway.

In recent years, several drugs that inhibit one or more components of the PI3K signaling pathway have been developed namely pan-PI3K inhibitors, isoform-specific PI3K inhibitors, double PI3K/mTOR inhibitors, mTORC1/2 and AKT inhibitors (25, 26). These agents have been evaluated in the different subtypes of breast cancer (luminal, triple-negative, HER2+ tumors) thereby obtaining the greatest benefits when combined with standard therapies and in tumors presenting an hyperactivation of the PI3K signaling pathway (mostly due to *PIK3CA* activating mutations/amplification, inactivating mutations or loss of PTEN, or AKT activating mutations). In the HER2+ breast cancer subtype, preclinical evidence has shown that inhibitors of the PI3K/AKT/mTOR pathway can enhance the antiproliferative activity of anti-HER2 therapies and/or overcome the acquired resistance to these agents (9, 27–33). Among these, PI3K pan-inhibitors, buparlisib and taselisib have been shown to be particularly active in HER2+ breast cancer models with *PIK3CA* activating mutations (11). However, the clinical developments of such agents has been precluded by their toxicity profiles which may be partly explained by the lack of selectivity towards the different catalytic isoforms of the class IA PI3K (p110α, β, γ and δ). In mammals, p110α and p110β that are encoded by the *PIK3CA* and *PIK3CB* genes are expressed in all cell types, whereas p110δ and p110γ encoded by the *PIK3CD* and *PIK3CG* genes are found mainly in hematopoietic cells. Of the four PI3K catalytic subunits, p110α is the most frequently mutated isoform in cancers. Indeed, it is the major effector downstream of the receptor tyrosine kinases, and plays a prominent role in controlling cell growth and proliferation (34). However, the other catalytic isoforms of PI3K also play an important role in cell growth, metabolism and tumorigenesis (35, 36). Interestingly, the finding that p110α inhibition leads to reactivation of PI3K signaling *via* p110β suggests that a concomitant inhibition of both isoforms serves to more efficiently block PI3K-dependent oncogenic signaling in HER2+ breast cancer models (35, 36). Alpelisib is an α-specific class I PI3K inhibitor that inhibits the phosphorylation of downstream PI3K target proteins, including AKT, and it also inhibits the growth of *PIK3CA* mutant breast cancer cell lines *in vitro* and *in vivo*. Alpelisib received Food and Drug Administration and European Medicines Agency approval based on SOLAR-1, a phase III, randomized, double-blinded, placebo-controlled trial that evaluates the efficacy and toxicity profile of alpelisib or placebo in combination with fulvestrant in 572 postmenopausal patients with HR+/HER2- advanced breast cancer whose disease had progressed or relapsed during or after treatment with an aromatase inhibitor (30). Among them, 341 had *PIK3CA*-activating mutations. The SOLAR-1 study demonstrated that, in the cohort of patients with mutated *PIK3CA*, the addition of alpelisib to fulvestrant significantly prolonged progression-free survival (estimated 35% reduction in the risk of disease progression or death, median progression-free survival =11 and 5.7 months for alpelisib and placebo, respectively) while maintaining a profile of acceptable toxicity (37). To date, alpelisib is the only PI3K inhibitor approved for the treatment of breast cancer patients.

We hypothesize that the alpha-specific PI3K inhibitor alpelisib could be the best drug with which to target the PI3K pathway in order to enhance the antitumor efficacy of anti-HER2 therapy in HER2+/*PIK3CA* mutant breast cancer. We tested alpelisib in monotherapy or in combination with anti-HER2 drugs in preclinical models of HER2+ breast cancer and measured cell viability by MTT assay. In line with the results obtained with other PI3K inhibitors, we observed that alpelisib significantly inhibited cell growth and enhanced the effect of the anti-HER2 monoclonal antibodies trastuzumab and pertuzumab *in vitro*. In HER2+/*PIK3CA* mutated breast cancer cell lines, alpelisib was more potent than trastuzumab or trastuzumab + pertuzumab in inhibiting the *in vitro* growth and the combination of alpelisib plus trastuzumab also showed synergistic activity. The use of alternative techniques for measuring cell viability (38) along with additional *in vitro* tumor models are needed to confirm and extend these findings.

Western blot analysis of PI3K pathway components revealed that alpelisib plus anti-HER2 treatments were accompanied by a more pronounced decrease in p-S6K protein levels compared with alpelisib or anti-HER2 therapy alone in all HER2+ BC cell lines. However, while such combinations led to a higher suppression of p-AKT levels than alpelisib or anti-HER2 therapy alone in BT474 and SKBR3 cell lines, p-AKT levels did not differ between treatments in KPL4 and HCC1954 models. We hypothesized that differences in intracellular pathway signaling networks may explain, at least in part, the differences in terms of inhibition of PI3K pathway signaling nodes across our *PIK3CA* mutant cell lines. However, future studies using a broader range of alpelisib doses in combination with anti-HER2 therapy and a proteomic assay to extensively investigate signaling pathways are needed to verify this hypothesis. The antitumor efficacy of alpelisib was confirmed *in vivo*, where the addition of the alpha-selective PI3K inhibitor to trastuzumab significantly reduced the growth of HCC1954 cell xenografts. HCC1954 cells are estrogen- and progesterone receptors negative but express the androgen receptor (39). We recognize that the reduced levels of estrogen in the ovariectomized mice used for our *in vivo* experiment may have influenced androgen signaling which have been showed to promote breast tumor growth and metastasis through different mechanisms including the activation of the HER2-downstream PI3K/PTEN and ERK pathways (39, 40).

Overall, our data support the clinical development of alpelisib as part of therapeutic strategies for patients with HER2+ breast cancer harboring *PIK3CA* activating mutations. In this context the phase III, multicenter, randomized ALPHABET trial is investigating the efficacy and safety of alpelisib + trastuzumab ± fulvestrant versus trastuzumab + chemotherapy in patients with HER2+/*PIK3CA*-mutated advanced breast cancer progressing to a previous anti-HER2 treatment (NCT05063786).

A major limitation of targeted anticancer therapies is intrinsic or acquired resistance. Deciphering the molecular mechanisms underlying resistance offers the possibility to develop prognostic and predictive factors and to identify novel therapeutic targets to prevent and/or overcome resistance. Therefore, we generated models of acquired resistance to monotherapy with alpelisib and to the combination of alpelisib plus trastuzumab which we subsequently characterized by analyzing their genomic (DNA-targeted sequencing) and transcriptomics (RNA-sequencing) profiles. We identified diverse biological processes commonly hyperactivated in our resistant models (oxidation-reduction, cell proliferation and division, immune response, RNA synthesis) and gene candidates potentially responsible for promoting alpelisib resistance. The latter include the *AKR1C1* and *AKR1C2* genes, which belong to the AKR1C aldo-keto reductase family (41). This family consists of four enzymes (AKR1C1-4) that catalyze NADPH-dependent reductions and performs important functions in the processes of biosynthesis, intermediate metabolism and detoxification (41). Recent studies have shown a strong correlation between the expression levels of the AKR1C family members and neoplastic transformation, tumor invasion and resistance to cancer therapies (42–44). A search of publicly available databases showed that the expression of the *AKR1C1* and *AKR1C2* genes is inversely correlated with the *in vitro* sensitivity to alpelisib in a large panel of breast cancer cell lines. Furthermore, a high expression of *AKR1C1*, but not of *AKR1C2* had a negative prognostic value in patients with HER2+ early stage breast tumors. We also found that inhibition of *AKR1C1* mRNA levels using siRNA resulted in a greater inhibition of cell proliferation in models resistant to alpelisib (HCC1954 AR and KPL4 AR) versus the corresponding parental cells (HCC1954 and KPL4). Overall, these data support the potential role of *AKR1C1* and *AKR1C2* as determinants of resistance to alpelisib. In this study, we also observed a significant association between high expression of the small nuclear ribonucleoprotein polypeptide G (*SNRPG*) gene and improved relapse-free survival in patients with HER2+ early stage breast tumors. *SNRPG* encoded for the Smith protein G that is involved in the biogenesis of spliceosomal uridyl-rich small nuclear ribonucleoprotein particles (45). The biological role of *SNRPG* in solid tumors is still unclear (45, 46) and further studies are needed to assess its value as a prognostic marker and/or a therapeutic target in breast cancer.

In conclusion, the results of our study confirm the negative impact of *PIK3CA* mutations on the efficacy of the anti-HER2 monoclonal antibodies, trastuzumab and pertuzumab that are used to treat patients with HER2+ breast cancer and support the clinical development of the alpha-selective PI3K inhibitor alpelisib to enhance their antitumor activity. The integration of omics data arising from our preclinical models of resistance to alpelisib and future experiments will clarify the mechanistic aspects underlying this resistance. This approach will enable us to identify biomarkers with which to select patients with HER2+ breast cancer who would most benefit from treatment with alpelisib. It may also lead to new therapeutic targets for the development of new therapeutic strategies.

## Data availability statement

The raw data supporting the conclusions of this article will be made available by the authors, without undue reservation.

## Ethics statement

The animal study was reviewed and approved by University Federico II Veterinary Ethical Committee.

## Author contributions

Conceptualization: MC, CD, BV. Methodology: MC, CD, BV. Software and formal analysis: MC, PD, DE, LF, CD. Investigation: MC, PD, DE, LF, GA, MG: Resources. CD, RB, BV. Data curation: MC, PD, DE, LF, CD. Writing—review and editing: MC, CD, BV: Supervision. BV: Funding acquisition. CD, RB, BV. All authors contributed to the article and approved the submitted version.

## Supplementary material

Supplementary Table 1 Differentially Expressed Genes (DEGs) in alpelisib-resistant vs -sensitive HER2 +/*PIK3CA* mutant breast cancer cell lines. Comparisons of gene expression between alpelisib-resistant (AR) and alpelisib+trastuzumab-resistant (ATR) HCC1954 and KPL4 derivatives vs parental cells treated with alpelisib (+A) or alpelisib+trastuzumab (+AT) for 48 hours were performed using DESeq2. The Wald test was used to generate p-values and log2 fold changes. Genes with an adjusted p-value < 0.05 and absolute log2 fold change > 1 were defined as DEGs.

## References

[1] Martínez-Sáez O Prat A. Current and future management of HER2-positive metastatic breast cancer. JCO Oncol Pract (2021) 17:594–604. doi: 10.1200/OP.21.00172 34077236

[2] Veeraraghavan J De Angelis C Reis-Filho JS Pascual T Prat A Rimawi MF. De-escalation of treatment in HER2-positive breast cancer: determinants of response and mechanisms of resistance. Breast (2017) 34 Suppl:1, S19–S26. doi: 10.1016/j.breast.2017.06.022 PMC6050048 28687441

[3] Rimawi MF De Angelis C Schiff R. Resistance to anti-HER2 therapies in breast cancer. Am Soc Clin Oncol Educ Book (2015), e157–64. doi: 10.14694/EdBook_AM.2015.35.e157 25993167

[4] Xu X De Angelis C Burke KA Nardone A Hu H Qin L. HER2 reactivation through acquisition of the HER2 L755S mutation as a mechanism of acquired resistance to HER2-targeted therapy in HER2+ breast cancer. Clin Cancer Res (2017) 23:5123–34. doi: 10.1158/1078-0432.CCR-16-2191 PMC5762201 28487443

[5] Goutsouliak K Veeraraghavan J Sethunath V De Angelis C Osborne CK Rimawi MF. Towards personalized treatment for early stage HER2-positive breast cancer. Nat Rev Clin Oncol (2020) 17:233–50. doi: 10.1038/s41571-019-0299-9 PMC8023395 31836877

[6] Baselga J Cortés J Im SA Clark E Ross G Kiermaier A. Biomarker analyses in CLEOPATRA: a phase III, placebo-controlled study of pertuzumab in human epidermal growth factor receptor 2-positive, first-line metastatic breast cancer. J Clin Oncol (2014) 32:3753–61. doi: 10.1200/JCO.2013.54.5384 25332247

[7] Rimawi MF De Angelis C Contreras A Pareja F Geyer FC Burke KA. Low PTEN levels and PIK3CA mutations predict resistance to neoadjuvant lapatinib and trastuzumab without chemotherapy in patients with HER2 over-expressing breast cancer. Breast Cancer Res Treat (2018) 167:731–40. doi: 10.1007/s10549-017-4533-9 PMC5821069 29110152

[8] Veeraraghavan J De Angelis C Mao R Wang T Herrera S Pavlick AC. A combinatorial biomarker predicts pathologic complete response to neoadjuvant lapatinib and trastuzumab without chemotherapy in patients with HER2+ breast cancer. Ann Oncol (2019) 30:927–33. doi: 10.1093/annonc/mdz076 PMC6594453 30903140

[9] Berns K Horlings HM Hennessy BT Madiredjo M Hijmans EM Beelen K. A functional genetic approach identifies the PI3K pathway as a major determinant of trastuzumab resistance in breast cancer. Cancer Cell (2007) 12:395–402. doi: 10.1016/j.ccr.2007.08.030 17936563

[10] Loibl S Majewski I Guarneri V Nekljudova V Holmes E Bria E. PIK3CA mutations are associated with reduced pathological complete response rates in primary HER2-positive breast cancer: pooled analysis of 967 patients from five prospective trials investigating lapatinib and trastuzumab. Ann Oncol (2016) 27:1519–25. doi: 10.1093/annonc/mdw197 PMC6279074 27177864

[11] Utermark T Rao T Cheng H Wang Q Lee SH Wang ZC. The p110α and p110β isoforms of PI3K play divergent roles in mammary gland development and tumorigenesis. Genes Dev (2012) 26:1573–86. doi: 10.1101/gad.191973.112 PMC3404385 22802530

[12] Hanker AB Pfefferle AD Balko JM Kuba MG Young CD Sánchez V. Mutant PIK3CA accelerates HER2-driven transgenic mammary tumors and induces resistance to combinations of anti-HER2 therapies. Proc Natl Acad Sci USA (2013) 110:14372–7. doi: 10.1073/pnas.1303204110 PMC3761610 23940356

[13] Fuso P Muratore M D'Angelo T Paris I Carbognin L Tiberi G. PI3K inhibitors in advanced breast cancer: the past, the present, new challenges and future perspectives. Cancers (Basel) (2022) 14(9):2161. doi: 10.3390/cancers14092161 35565291 PMC9103982

[14] Mayer IA Arteaga CL. The PI3K/AKT pathway as a target for cancer treatment. Annu Rev Med (2016) 67:11–28. doi: 10.1146/annurev-med-062913-051343 26473415

[15] Janku F Yap TA Meric-Bernstam F. Targeting the PI3K pathway in cancer: are we making headway? Nat Rev Clin Oncol (2018) 15:273–91. doi: 10.1038/nrclinonc.2018.28 29508857

[16] Loibl S de la Pena L Nekljudova V Zardavas D Michiels S Denkert C. Neoadjuvant buparlisib plus trastuzumab and paclitaxel for women with HER2+ primary breast cancer: a randomised, double-blind, placebo-controlled phase II trial (NeoPHOEBE). Eur J Cancer (2017) 85:133–45. doi: 10.1016/j.ejca.2017.08.020 PMC5640494 28923573

[17] Montanari M Carbone MR Coppola L Giuliano M Arpino G Lauria R. Epigenetic silencing of THY1 tracks the acquisition of the Notch1-EGFR signaling in a xenograft model of CD44+/CD24low/CD90+ myoepithelial cells. Mol Cancer Res (2019) 17:628–41. doi: 10.1158/1541-7786.MCR-17-0324 30242055

[18] Veneziani BM Criniti V Cavaliere C Corvigno S Nardone A Picarelli S. *In vitro* expansion of human breast cancer epithelial and mesenchymal stromal cells: optimization of a coculture model for personalized therapy approaches. Mol Cancer Ther (2007) 6:3091–100. doi: 10.1158/1535-7163.MCT-07-0356 18089705

[19] De Angelis C Fu X Cataldo ML Nardone A Pereira R Veeraraghavan J. Activation of the IFN signaling pathway is associated with resistance to CDK4/6 inhibitors and immune checkpoint activation in ER-positive breast cancer. Clin Cancer Res (2021) 27:4870–82. doi: 10.1158/1078-0432.CCR-19-4191 PMC8628647 33536276

[20] Young MD Wakefield MJ Smyth GK Oshlack A. Gene ontology analysis for RNA-seq: accounting for selection bias. Genome Biol (2010) 11:R14. doi: 10.1186/gb-2010-11-2-r14 20132535 PMC2872874

[21] Györffy B Lanczky A Eklund AC Denkert C Budczies J Li Q. An online survival analysis tool to rapidly assess the effect of 22,277 genes on breast cancer prognosis using microarray data of 1,809 patients. Breast Cancer Res Treat (2010) 123:725–31. doi: 10.1007/s10549-009-0674-9 20020197

[22] Hather G Liu R Bandi S Mettetal J Manfredi M Shyu WC. Growth rate analysis and efficient experimental design for tumor xenograft studies. Cancer Inform (2014) 13:65–72. doi: 10.4137/CIN.S13974 25574127 PMC4264612

[23] Chou T-C. Drug combination studies and their synergy quantification using the chou-talalay method. Cancer Res (2010) 70:440–6. doi: 10.1158/0008-5472.CAN-09-1947 20068163

[24] Bianchini G Kiermaier A Bianchi GV Im YH Pienkowski T Liu MC. Biomarker analysis of the NeoSphere study: pertuzumab, trastuzumab, and docetaxel versus trastuzumab plus docetaxel, pertuzumab plus trastuzumab, or pertuzumab plus docetaxel for the neoadjuvant treatment of HER2-positive breast cancer. Breast Cancer Res (2017) 19:16. doi: 10.1186/s13058-017-0806-9 28183321 PMC5299741

[25] Hoxhaj G Manning BD. The PI3K-AKT network at the interface of oncogenic signalling and cancer metabolism. Nat Rev Cancer (2020) 20:74–88. doi: 10.1038/s41568-019-0216-7 31686003 PMC7314312

[26] Ortega MA Fraile-Martínez O Asúnsolo Á Buján J García-Honduvilla N Coca S. Signal transduction pathways in breast cancer: the important role of PI3K/Akt/mTOR. J Oncol (2020) 2020:9258396. doi: 10.1155/2020/9258396 32211045 PMC7085392

[27] Eichhorn PJA Gili M Scaltriti M Serra V Guzman M Nijkamp W. Phosphatidylinositol 3-kinase hyperactivation results in lapatinib resistance that is reversed by the mTOR/phosphatidylinositol 3-kinase inhibitor NVP-BEZ235. Cancer Res (2008) 68:9221–30. doi: 10.1158/0008-5472.CAN-08-1740 PMC2587064 19010894

[28] García-García C Ibrahim YH Serra V Calvo MT Guzmán M Grueso J. Dual mTORC1/2 and HER2 blockade results in antitumor activity in preclinical models of breast cancer resistant to anti-HER2 therapy. Clin Cancer Res (2012) 18:2603–12. doi: 10.1158/1078-0432.CCR-11-2750 22407832

[29] Chakrabarty A Sánchez V Kuba MG Rinehart C Arteaga CL. Feedback upregulation of HER3 (ErbB3) expression and activity attenuates antitumor effect of PI3K inhibitors. Proc Natl Acad Sci USA (2012) 109:2718–23. doi: 10.1073/pnas.1018001108 PMC3286932 21368164

[30] Serra V Scaltriti M Prudkin L Eichhorn PJ Ibrahim YH Chandarlapaty. PI3K inhibition results in enhanced HER signaling and acquired ERK dependency in HER2-overexpressing breast cancer. Oncogene (2011) 30:2547–57. doi: 10.1038/onc.2010.626 PMC3107390 21278786

[31] Rexer BN Chanthaphaychith S Dahlman K Arteaga CL. Direct inhibition of PI3K in combination with dual HER2 inhibitors is required for optimal antitumor activity in HER2+ breast cancer cells. Breast Cancer Res (2014) 16:R9. doi: 10.1186/bcr3601 24451154 PMC3978602

[32] O’Brien NA Browne BC Chow L Wang Y Ginther C Arboleda J. Activated phosphoinositide 3-kinase/AKT signaling confers resistance to trastuzumab but not lapatinib. Mol Cancer Ther (2010) 9:1489–502. doi: 10.1158/1535-7163.MCT-09-1171 20501798

[33] Fujimoto Y Morita TY Ohashi A Haeno H Hakozaki Y Fujii M. Combination treatment with a PI3K/Akt/mTOR pathway inhibitor overcomes resistance to anti-HER2 therapy in PIK3CA-mutant HER2-positive breast cancer cells. Sci Rep (2020) 10:21762. doi: 10.1038/s41598-020-78646-y 33303839 PMC7729878

[34] Fruman DA Chiu H Hopkins BD Bagrodia S Cantley LC Abraham RT. The PI3K pathway in human disease. Cell (2017) 170:605–35. doi: 10.1016/j.cell.2017.07.029 PMC5726441 28802037

[35] Jia S Liu Z Zhang S Liu P Zhang L Lee SH. Essential roles of PI(3)K-p110beta in cell growth, metabolism and tumorigenesis. Nature (2008) 454:776–9. doi: 10.1038/nature07091 PMC2750091 18594509

[36] Thorpe LM Yuzugullu H Zhao JJ. PI3K in cancer: divergent roles of isoforms, modes of activation and therapeutic targeting. Nat Rev Cancer (2015) 15:7–24. doi: 10.1038/nrc3860 25533673 PMC4384662

[37] André F Ciruelos E Rubovszky G Campone M Loibl S Rugo HS. Alpelisib for PIK3CA-mutated, hormone receptor-positive advanced breast cancer. N Engl J Med (2019) 380:1929–40. doi: 10.1056/NEJMoa1813904 31091374

[38] Riss TL Moravec RA Niles AL Duellman S Benink HA Worzella TJ. Assay guidance manual [Internet]. Bethesda (MD): Eli Lilly & Company and the National Center for Advancing Translational Sciences. (2004). 22553861

[39] He L Du Z Xiong X Ma H Zhu Z Gao H. Targeting androgen receptor in treating HER2 positive breast cancer. Sci Rep (2017) 7:14584. doi: 10.1038/s41598-017-14607-2 29109513 PMC5674043

[40] Michmerhuizen AR Spratt DE Pierce LJ Speers CW. ARe we there yet? understanding androgen receptor signaling in breast cancer. NPJ Breast Cancer (2020) 6:47. doi: 10.1038/s41523-020-00190-9 33062889 PMC7519666

[41] Zeng C-M Chang LL Ying MD Cao J He QJ Zhu H. Aldo-keto reductase AKR1C1-AKR1C4: functions, regulation, and intervention for anti-cancer therapy. Front Pharmacol (2017) 8:119. doi: 10.3389/fphar.2017.00119 28352233 PMC5349110

[42] Shirato A Kikugawa T Miura N Tanji N Takemori N Higashiyama S. Cisplatin resistance by induction of aldo-keto reductase family 1 member C2 in human bladder cancer cells. Oncol Lett (2014) 7:674–8. doi: 10.3892/ol.2013.1768 PMC3919892 24527071

[43] Bortolozzi R Bresolin S Rampazzo E Paganin M Maule F Mariotto E. AKR1C enzymes sustain therapy resistance in paediatric T-ALL. Br J Cancer (2018) 118:985–94. doi: 10.1038/s41416-018-0014-0 PMC5931104 29515258

[44] Matsumoto R Tsuda M Yoshida K Tanino M Kimura T Nishihara H. Aldo-keto reductase 1C1 induced by interleukin-1β mediates the invasive potential and drug resistance of metastatic bladder cancer cells. Sci Rep (2016) 6:34625. doi: 10.1038/srep34625 27698389 PMC5048132

[45] Mabonga L Kappo AP. The oncogenic potential of small nuclear ribonucleoprotein polypeptide G: a comprehensive and perspective view. Am J Transl Res (2019) 11:6702–16. PMC6895504 31814883

[46] Lan Y Lou J Hu J Yu Z Lyu W Zhang B. Downregulation of SNRPG induces cell cycle arrest and sensitizes human glioblastoma cells to temozolomide by targeting myc through a p53-dependent signaling pathway. Cancer Biol Med (2020) 17:112–31. doi: 10.20892/j.issn.2095-3941.2019.0164 PMC7142844 32296580

