# Supplementary material for: The effect of the alpha-specific PI3K inhibitor alpelisib combined with anti-HER2 therapy in HER2+/PIK3CA mutant breast cancer
Source: Front Oncol. 2023 Jul 4;13:1108242. doi: 10.3389/fonc.2023.1108242 (PMC10353540; doi:10.3389/fonc.2023.1108242)

Supplementary Figure S1. Bar chart representing the functionally enriched gene ontology terms associated with resistance to alpelisib (AR) and alpelisib + trastuzumab (ATR) in HER2+/PIK3CA mutant breast cancer cell lines.

- **Gene ontology enrichment in KPL4 AR versus KPL4 treated with alpelisib for 48 hours**

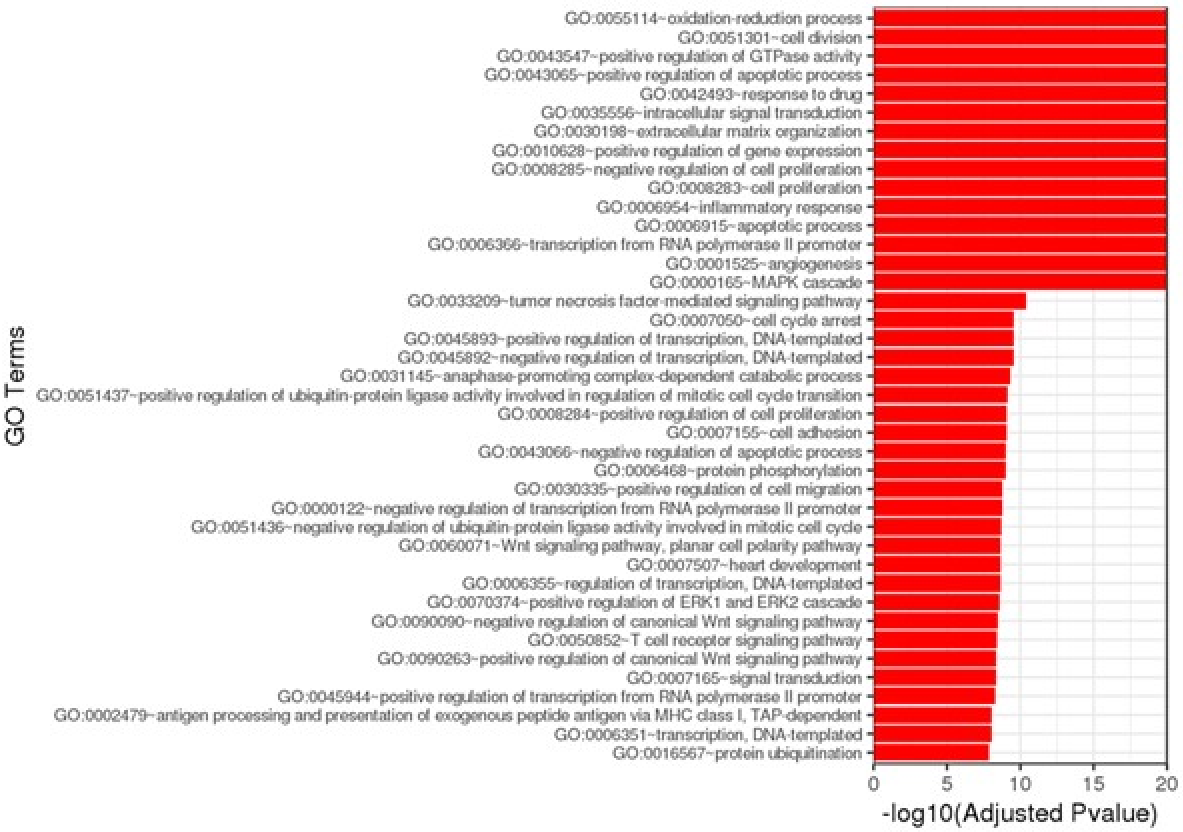

- **Gene ontology enrichment in KPL4 ATR versus KPL4 treated with alpelisib plus trastuzumab for 48 hours**

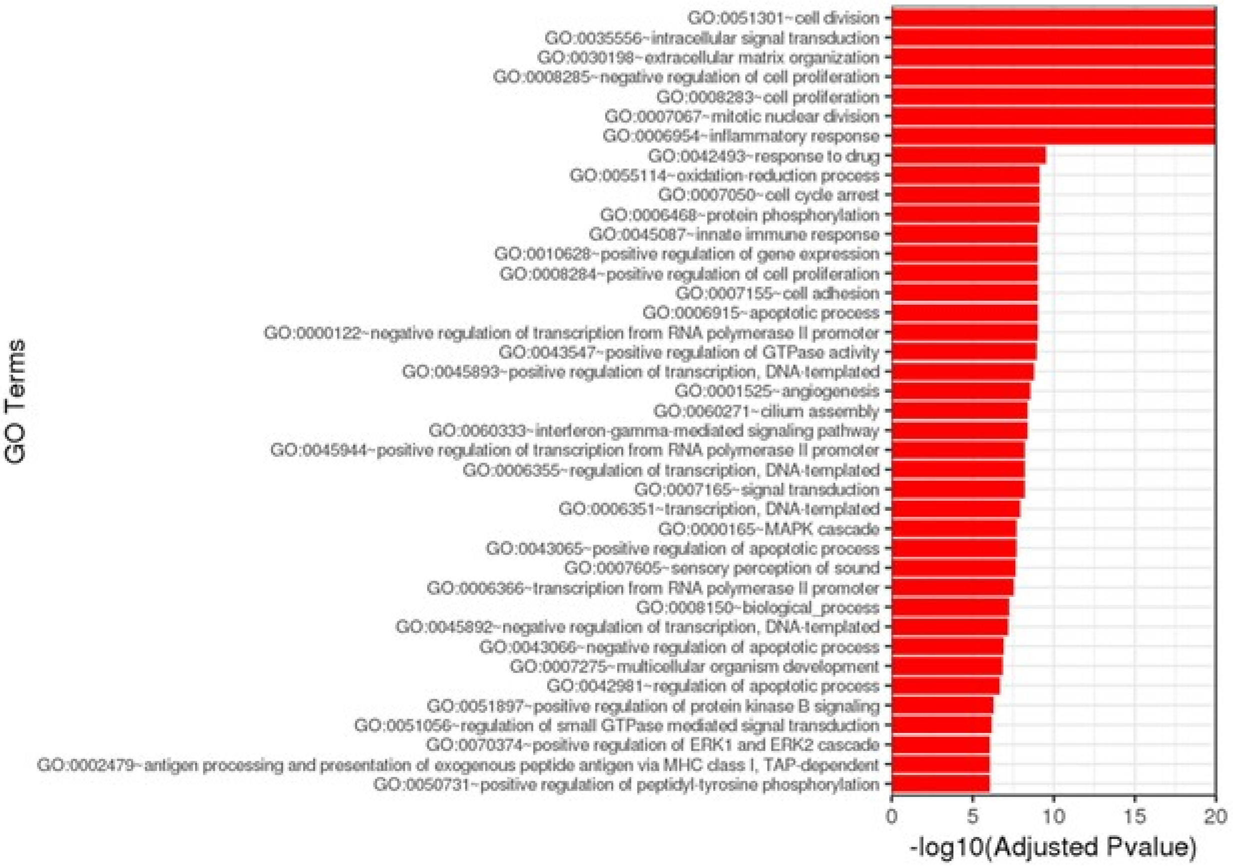

- **Gene ontology enrichment in HCC1954 AR versus Hcc1954 treated with alpelisib for 48 hours**

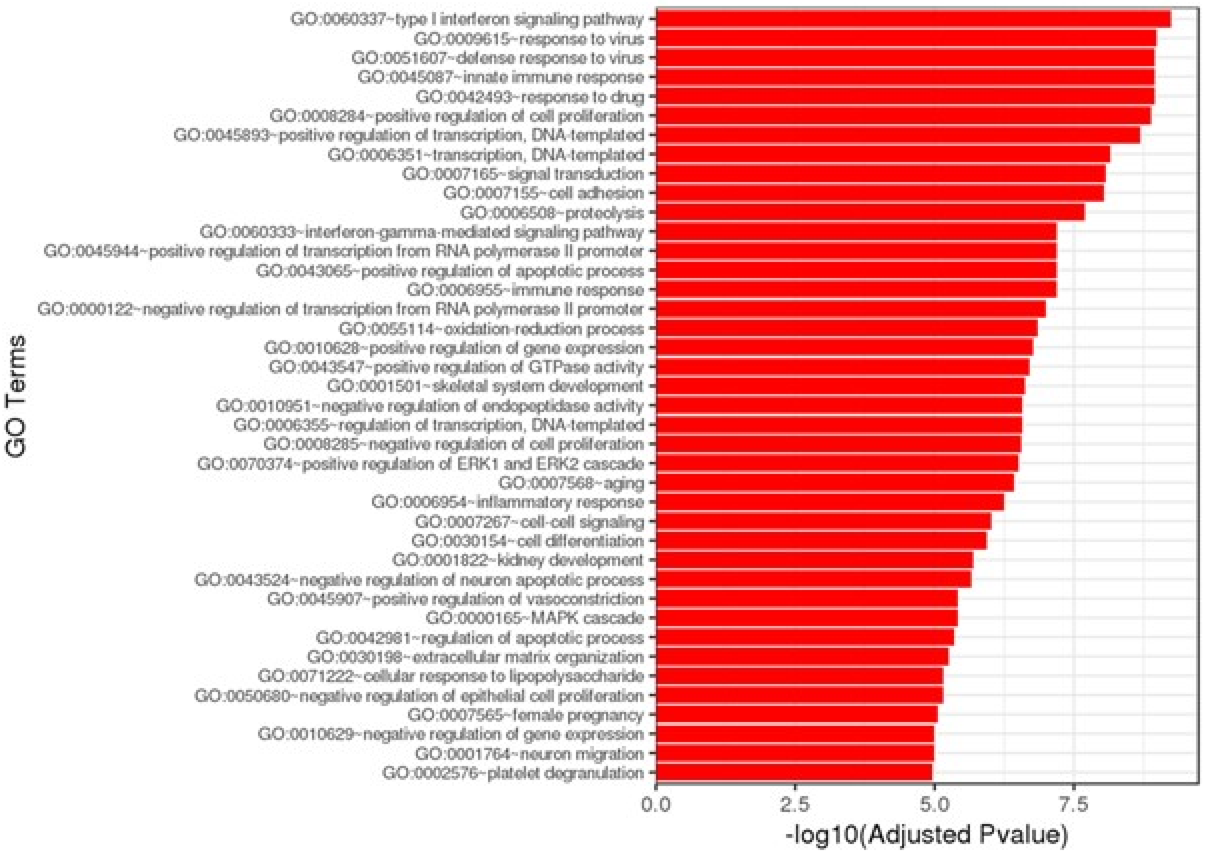

- **Gene ontology enrichment in HCC1954 ATR versus HCC1954 treated with alpelisib plus trastuzumab for 48 hours**

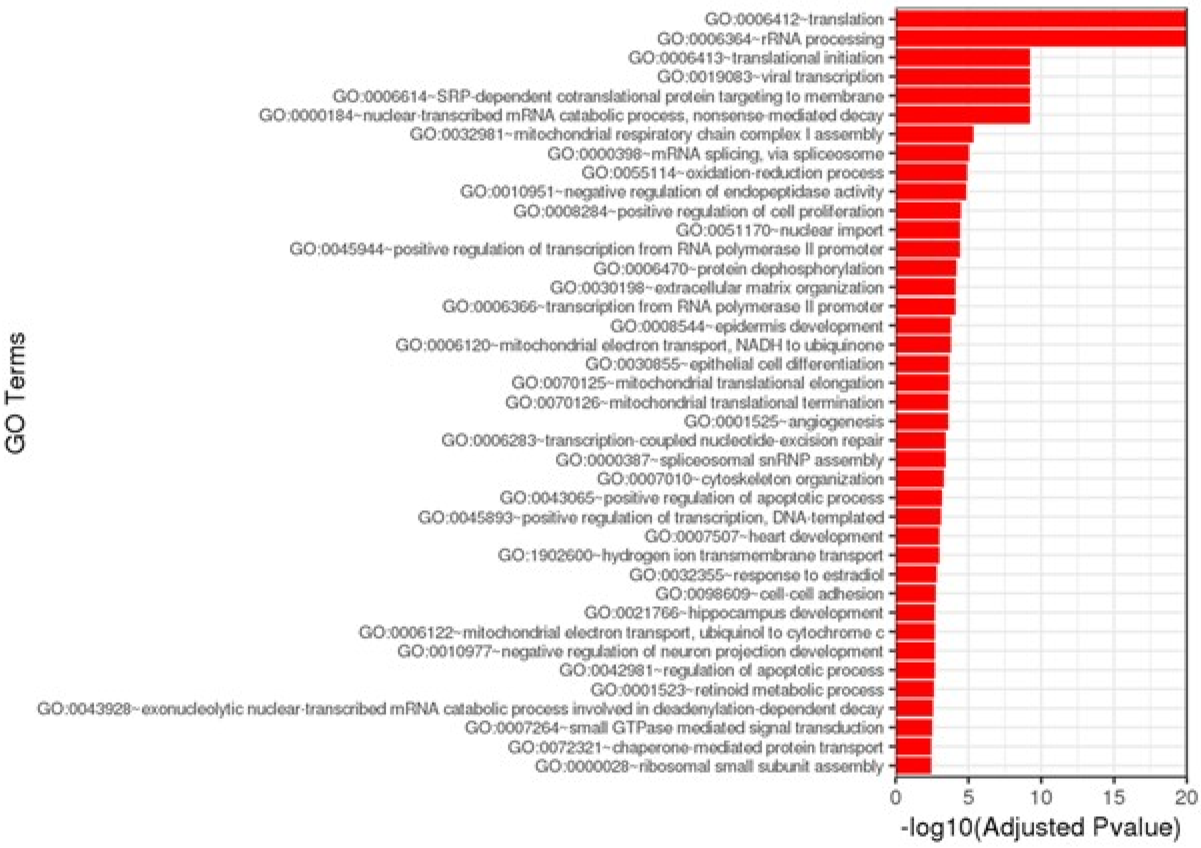

Supplement: Supplementary file 1 [file Image_1.pdf]
