# Supplementary figures and images for: The effect of the alpha-specific PI3K inhibitor alpelisib combined with anti-HER2 therapy in HER2+/PIK3CA mutant breast cancer
Source: Front Oncol. 2023 Jul 4;13:1108242. doi: 10.3389/fonc.2023.1108242 (PMC10353540; doi:10.3389/fonc.2023.1108242)

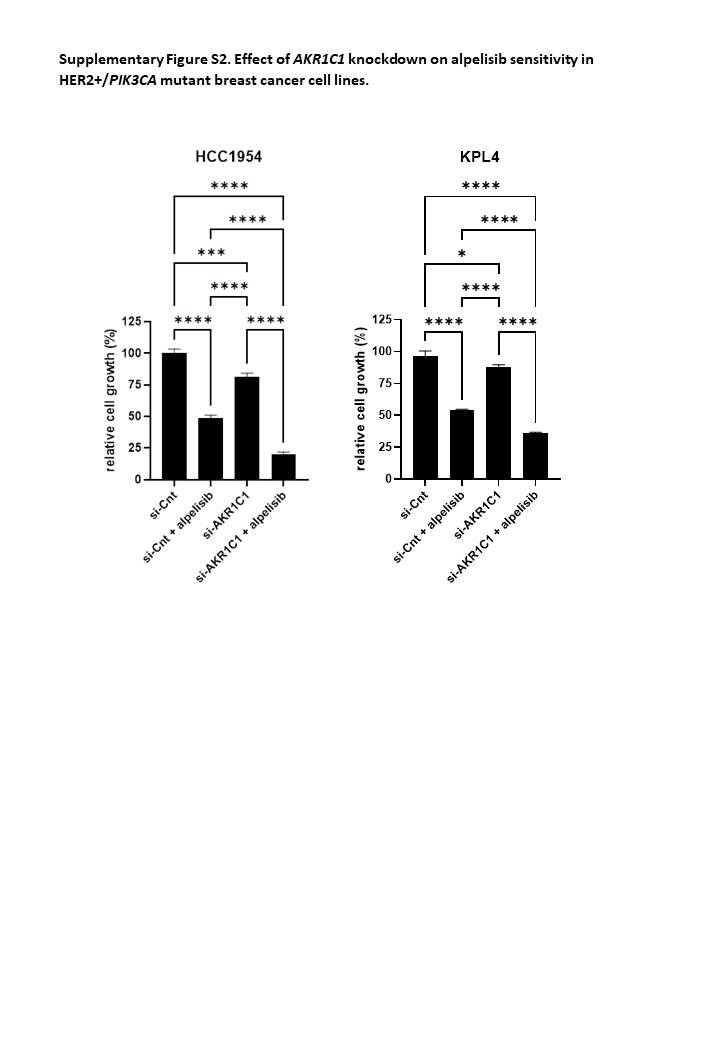

Supplement: Supplementary file 2 [file Image_2.jpeg]
